# Identification and Functional Analysis of Cytokine-Like Protein CLEC-47 in Caenorhabditis elegans

**DOI:** 10.1128/mBio.02579-21

**Published:** 2021-10-12

**Authors:** Wen Pan, Xiaowen Huang, Zeyuan Guo, Rekha Nagarajan, Eleftherios Mylonakis

**Affiliations:** a Division of Infectious Diseases, Rhode Island Hospital, Warren Alpert Medical School of Brown University, Providence, Rhode Island, USA; b Department of Dermatology, Nanfang Hospital, Southern Medical University, Guangzhou, China; c Department of Molecular Pharmacology, Physiology, and Biotechnology at Brown University, Providence, Rhode Island, USA; Harvard Medical School

**Keywords:** *Caenorhabditis elegans*, CLEC-47, cytokine, C-type lectin domain-containing proteins

## Abstract

A variety of effector proteins contribute to host defense in Caenorhabditis elegans. However, beyond lytic enzymes and antimicrobial peptides and proteins, little is known about the exact function of these infection-related effectors. This study set out to identify pathogen-dependent cytokine-like molecules, focusing on C-type lectin domain-containing proteins (CLECs). In total, 38 CLECs that are differentially regulated in response to bacterial infections have been previously identified by microarray and transcriptome sequencing (RNA-seq) analyses in C. elegans. We successfully cloned 18 of these 38 CLECs and chose to focus on CLEC-47 because, among these 18 cloned CLECs, it was the smallest protein and was recombinantly expressed at the highest levels in prokaryotic cells examined by SDS-PAGE. Quantitative real-time PCR (qRT-PCR/qPCR) showed that the expression of *clec-47* was induced by a variety of Gram-positive bacterial pathogens, including Enterococcus faecium, Staphylococcus aureus, and Cutibacterium acnes, but was suppressed by the Gram-negative bacteria Klebsiella pneumoniae and Pseudomonas aeruginosa. By expressing CLEC-47 in HEK 293 cells, we showed that CLEC-47 is released into the culture media, which the Golgi apparatus inhibitors (brefeldin A [BFA] and GolgiStop) could block. Purified recombinant CLEC-47 (maltose binding protein [MBP]–CLEC-47–His) did not display antimicrobial activity against ESKAPE pathogen isolates but bound directly to murine macrophage J774A.1 cells. Recombinant CLEC-47 attracted and recruited J774A.1 cells in a chemotaxis assay. In addition, qPCR studies and enzyme-linked immunosorbent assays (ELISAs) showed that CLEC-47 activates J774A.1 cells in a dose- and time-dependent manner to express the proinflammatory cytokines tumor necrosis factor alpha (TNF-α), interleukin-1β (IL-1β), IL-6, and Macrophage Inflammatory Protein 2 (MIP-2). Moreover, C. elegans, fed with CLEC-47-expressing Escherichia coli, demonstrated enhanced expression of several antimicrobial proteins (CNC-1, CNC-2, CPR-1, and CPR-2) as well as the detoxification protein MTL-1. These data suggest that CLEC-47 functions as a novel cytokine-like signaling molecule and exemplify how the study of infection-related effectors in C. elegans can help elucidate the evolution of immune responses.

## INTRODUCTION

The well-studied nematode Caenorhabditis elegans is a model host that consumes microbes as a food source (1). Nematodes have developed a complex defense mechanism to resist bacterial infection, which is characterized by the production of infection-related effectors (2, 3) such as reactive oxygen species and antimicrobial peptides and proteins (including lysozymes, caenopores, defensin-like peptides, caenacins, and neuropeptide-like proteins) (4). C-type lectin domain-containing (CLEC) proteins and CUB_2 domain-containing proteins are the major putative effector families identified by transcriptional profiling (5–10). However, to date, there are only limited reports on the functions of the vast majority of C. elegans immune effectors, with the exception of some antimicrobial peptides and lytic proteins (11).

Cytokines are secretory molecules that were first identified as key components of the vertebrate immune response, which mediate and regulate host immunity (12). Cytokines are produced in response to an immune stimulus, such as a signal of microbial invasion, tissue destruction, or the release of inflammatory factors (12, 13). Moreover, sets of cytokines function in networks to regulate each other to achieve immunostasis (14, 15). In brief, during infection of vertebrates, proinflammatory cytokines elicit inflammatory responses and stimulate the host to produce effectors (reactive oxygen species and antimicrobial peptides and proteins, etc.) that attack invading pathogens (16, 17). Proinflammatory cytokines include tumor necrosis factor alpha (TNF-α), interferon gamma (IFN-γ), interleukin-1α (IL-1α), IL-1β, IL-6, and the chemokine IL-8 (18). Meanwhile, anti-inflammatory cytokines such as IL-4, IL-10, and transforming growth factor β (TGF-β) downregulate inflammation and reduce damage in the host (19). Beyond immune cells, cytokines are also produced by immune-related cells such as endothelial cells, epithelial cells, fibroblasts, and stromal cells (20, 21).

Nematodes have pathogen-specific innate immune responses (22), and many C. elegans effectors do not directly exhibit antimicrobial activity, even though they appear to be actively involved in host defense during infection (23). Because some features of the innate immune response such as the p38 mitogen-activated protein kinase (MAPK) signaling pathway have been conserved between C. elegans and vertebrates, we reasoned that putative C. elegans immune signaling molecules might elicit specific immune responses in vertebrates (24). We therefore tested the hypothesis that C. elegans expresses evolutionarily conserved immune mediators that can be identified by their cytokine-like activity on mammalian immune cells. CLEC proteins in mammals have a wide range of functions, including a role in the immune response to pathogens (25–27). Here, we report that the C. elegans CLEC protein family member CLEC-47 exhibits cytokine-like activity in a murine macrophage cell line and induces antimicrobial proteins and a detoxification protein in worms.

## RESULTS

### Bioinformatic analysis of C. elegans CLEC proteins.

To determine the number of C-lectin-type genes in the C. elegans genome, we sequentially searched every possible C-type lectin gene (*clec*) in the WormBase database (https://www.wormbase.org) starting from *clec-1*. We identified 240 *clec* protein-encoding genes in the C. elegans strain N2 genome. After obtaining the CLEC protein sequences from the UniProt database, we analyzed the existence of transmembrane domains and signal peptides in each CLEC protein using the SignalP and TMHMM servers.

As shown in Table S1 in the supplemental material, only 25 C. elegans CLECs contain predicted transmembrane domains. Among the remaining CLEC proteins, 12 do not contain either a signal peptide or a transmembrane domain, suggesting that they are cytosolic in nematode cells (Table S2). The majority of CLECs in C. elegans are potentially secretory proteins that contain a signal peptide (Table S3). The high ratio of secretory (203 out of 240; 84.6%) to nonsecretory (37 out of 240; 15.4%) proteins suggests that secreted CLECs may play critical immune-related roles in nematodes.

10.1128/mBio.02579-21.6 TABLE S1 List of predicted transmembrane proteins. Download Table S1, XLSX file, 0.03 MB. Copyright © 2021 Pan et al. 2021 Pan et al. https://creativecommons.org/licenses/by/4.0/ This content is distributed under the terms of the Creative Commons Attribution 4.0 International license.

10.1128/mBio.02579-21.7 TABLE S2 List of predicted cytosolic proteins. Download Table S2, XLSX file, 0.01 MB. Copyright © 2021 Pan et al. 2021 Pan et al. https://creativecommons.org/licenses/by/4.0/ This content is distributed under the terms of the Creative Commons Attribution 4.0 International license.

10.1128/mBio.02579-21.8 TABLE S3 List of predicted secreted proteins. Download Table S3, XLSX file, 0.1 MB. Copyright © 2021 Pan et al. 2021 Pan et al. https://creativecommons.org/licenses/by/4.0/ This content is distributed under the terms of the Creative Commons Attribution 4.0 International license.

Multiple research groups studied the transcriptional expression profiles of C. elegans upon bacterial infections (6–10). From those expression profiles, we identified 38 members of the *clec* gene family that are induced or suppressed during different bacterial infections (Table 1). Here, we refer to these 38 *clec* genes as “immunity related.” Among these 38 immunity-related *clec* genes, 97.4% (37 out of 38) are predicted secretory proteins.

**TABLE 1:** Infection-related *clec* genes in C. elegans^*a*^

Gene name(s) (sequence)	Induced/suppressed	Pathogen(s)	Protein length (aa)	Presence of signal peptide	Reference(s)
*clec-2* (B0454.7)	I	P. aeruginosa (G^−^)	411	+	8
*clec-3* (C41H7.7)	I	P. aeruginosa (G^−^)	409	+	8
*clec-4* (Y38E10A.5)	I	P. aeruginosa (G^−^)	425	+	8
*clec-10* (C03H5.1)	S	P. aeruginosa (G^−^)	415	+	7, 8
*clec-13* (H16D19.1), *clec-15* (T07D10.4)	I	*Microbacterium nematophilum* (G^+^)	413	+	9
*clec-17* (E03H4.10)	I	P. aeruginosa (G^−^)	416	+	8, 9
*clec-28* (F49A5.5)	S	P. aeruginosa (G^−^)	402	-	8
*clec-41* (B0365.6)	I	P. aeruginosa (G^−^)	545	+	7, 8
*clec-42* (F16H6.1)	I	P. aeruginosa (G^−^)	555	+	8
*clec-45* (F07C4.2)	I	P. aeruginosa (G^−^)	155	+	8
*clec-47* (T09F5.9)	I	P. aeruginosa (G^−^)	160	+	8
*clec-50* (W04E12.8)	I	Serratia marcescens (G^−^)	321	+	6
*clec-52* (B0218.8)	S	P. aeruginosa (G^−^)	308	+	8, 56
	I	S. aureus (G^+^)		+	56
*clec-60* (ZK666.6)	S	P. aeruginosa (G^−^)	406	+	8
	I	*M. nematophilum* (G^+^), S. aureus (G^+^)		+	9, 56
*clec-61* (ZK666.7)	I	*M. nematophilum* (G^+^)	403	+	9
*clec-62* (F35C5.5)	I	P. aeruginosa (G^−^)	389	+	7, 9
*clec-63* (F35C5.6)	I	Erwinia carotovora (G^−^), Photorhabdus luminescens (G^−^)	411	+	10
*clec-65* (F35C5.8)	I	P. aeruginosa (G^−^)	372	+	8
*clec-66* (F35C5.9)	I	P. aeruginosa (G^−^)	394	+	7, 8
*clec-67* (F56D6.2)	I	P. aeruginosa (G^−^)	467	+	7 – 9
*clec-68* (F56D6.1)	I	P. aeruginosa (G^−^)	462	+	7 – 9
*clec-70* (Y46C8AL.3)	I	*M. nematophilum* (G^+^), S. aureus (G^+^)	466	+	9, 56
*clec-71* (Y46C8AL.4)	I	P. aeruginosa (G^−^), S. aureus (G^+^)	467	+	8, 56
*clec-72* (Y46C8AL.5)	I	S. aureus (G^+^)	466	+	56
*clec-74* (Y46C8AL.8)	I	P. aeruginosa (G^−^)	467	+	8
*clec-82* (Y54G2A.8)	I	*M. nematophilum* (G^+^)	464	+	9
*clec-85* (Y54G2A.6)	I	S. marcescens (G^−^), P. aeruginosa (G^−^)	280	+	6, 8
*clec-86* (C54D1.2)	I	P. aeruginosa (G^−^)	166	+	7 – 9
*clec-122* (Y25C1A.3)	I	P. aeruginosa (G^−^)	605	+	8
*clec-143* (ZK673.9)	I	P. aeruginosa (G^−^)	400	+	8
*clec-163* (Y39A1B.1)	I	P. aeruginosa (G^−^)	385	+	8
*clec-166* (F38A1.5)	S	P. aeruginosa (G^−^)	466	+	7
*clec-173* (T26C12.6)	I	P. aeruginosa (G^−^)	187	+	8
*clec-174* (Y46C8AL.2)	I	P. aeruginosa (G^−^)	456	+	8
*clec-186* (ZK896.7)	I	P. aeruginosa (G^−^)	353	+	7, 8
*clec-187* (ZK896.6)	I	P. aeruginosa (G^−^)	295	+	7
*clec-227* (F08H9.5)	S	P. aeruginosa (G^−^)	381	+	7
*clec-265* (M02F4.7)	I	P. aeruginosa (G^−^)	322	+	8

a I, induced; S, suppressed; G^−^, Gram negative; G^+^, Gram positive.

**FIG 1:**
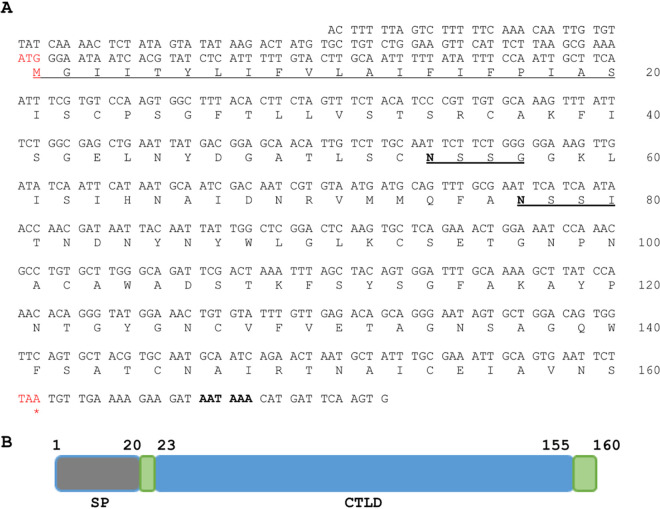
General information for C. elegans CLEC-47. (A) The nucleic acid and deduced amino acid sequences of CLEC-47. “ATG” in red indicates the start codon, “TAA” and “*” in red indicate the stop codon, AATAAA in boldface type at the 3′ end is the predicted poly(A) signal, capitalized letters with underlining at the N terminus indicate the signal peptide, and “N…” indicates the glycosylation site. (B) Schematic depiction of the conserved domains in CLEC-47. The predicted signal peptide (SP) is located at the N terminus. The C-type lectin-containing domain (CTLD) is located at aa 23 to 155.

To study the function of the 38 immunity-related *clec* genes, we attempted to clone as many of them as possible in Escherichia coli by amplifying their coding sequences from a C. elegans cDNA library. In total, 18/38 *clec* gene sequences were successfully cloned into the expression vector pET-51b without their signal sequences (Table 2). The pET-51b vector is designed for the high-level expression of recombinant proteins of interest fused to an N-terminal Strep-tag II and a C-terminal His tag (MilliporeSigma). Among the 18 successfully cloned *clec* genes, we chose *clec-47* for further analysis because it encodes the smallest protein (160 amino acids [aa]) (Fig. 1A).

**TABLE 2:** Molecular cloning of *clec* genes in C. elegans

Sequence	Gene name	Protein length (aa)	Mol wt (Da)	Restriction enzymes
C41H7.7	*clec-3*	409	45,626.86	KpnI/SacI
Y38E10A.5	*clec-4*	425	46,900.66	KpnI/SacI
H16D19.1	*clec-13*	413	45,156.63	KpnI/SacI
E03H4.10	*clec-17*	416	45,800.59	KpnI/SacI
T09F5.9	*clec-47*	160	17,130.4	KpnI/SacI
W04E12.8	*clec-50*	321	35,395.44	HindIII/NotI
B0218.8	*clec-52*	308	33,886.75	HindIII/NotI
ZK666.6	*clec-60*	406	44,065.18	KpnI/SacI
ZK666.7	*clec-61*	403	44,501.06	KpnI/NotI
F35C5.8	*clec-65*	372	41,626.56	KpnI/NotI
F35C5.9	*clec-66*	394	44,594.1	KpnI/SacI
F56D6.2	*clec-67*	467	51,690.58	KpnI/SacI
Y46C8AL.3	*clec-70*	466	52,034.96	BamHI/SacI
Y46C8AL.4	*clec-71*	467	52,977.45	KpnI/SacI
Y46C8AL.5	*clec-72*	466	51,986.36	KpnI/SacI
Y54G2A.6	*clec-85*	280	29,076.82	KpnI/NotI
C54D1.2	*clec-86*	166	18,483.81	KpnI/SacI
F08H9.5	*clec-227*	381	42,297.93	KpnI/SacI

### *clec-47* contains a signal sequence as well as a CLEC domain and displays diverse transcriptional responses to bacterial infection.

We analyzed the nucleic acid and amino acid sequences of CLEC-47 using the SignalP-5.0 (version 5.0) (Fig. S1) and TMHMM (version 2.0) (Fig. S2) servers and the Simple Modular Architecture Research Tool (SMART) (Fig. S3). As shown in Fig. 1A, we found a typical polyadenylation signal, AATAAA, which was located at the 3′ untranslated region (UTR) of the *clec-47* open reading frame. CLEC-47 has a theoretical molecular weight of 17.1 kDa and contains a common motif of signal sequences and a conserved C-type lectin domain (CTLD) (Fig. 1A). The signal sequence contains 20 amino acids at the N terminus of CLEC-47, which is a typical signal peptide with a likelihood probability of 0.9509 (Fig. S1). The CTLD is located between C23 and E155 (Fig. S3). After the removal of the signal sequence, the mature CLEC-47 has a single CTLD with a segment of 2 amino acids at the N terminus and a segment of 5 amino acids at the C terminus, which are displayed as two linkers (disordered regions) (Fig. 1B). This sequence distribution indicates that the function of CLEC-47 relies exclusively on the C-type lectin domain.

10.1128/mBio.02579-21.1 FIG S1 Prediction of the signal peptide of CLEC-47 by SignalP 5.0 (http://www.cbs.dtu.dk/services/SignalP/). Download FIG S1, XLSX file, 0.09 MB. Copyright © 2021 Pan et al. 2021 Pan et al. https://creativecommons.org/licenses/by/4.0/ This content is distributed under the terms of the Creative Commons Attribution 4.0 International license.

10.1128/mBio.02579-21.2 FIG S2 Prediction of the transmembrane domain of CLEC-47 by TMHMM (http://www.cbs.dtu.dk/services/TMHMM/). Download FIG S2, XLSX file, 0.01 MB. Copyright © 2021 Pan et al. 2021 Pan et al. https://creativecommons.org/licenses/by/4.0/ This content is distributed under the terms of the Creative Commons Attribution 4.0 International license.

10.1128/mBio.02579-21.3 FIG S3 Prediction of the conserved domain of CLEC-47 by SMART (http://smart.embl-heidelberg.de). Download FIG S3, XLSX file, 0.4 MB. Copyright © 2021 Pan et al. 2021 Pan et al. https://creativecommons.org/licenses/by/4.0/ This content is distributed under the terms of the Creative Commons Attribution 4.0 International license.

**FIG 2:**
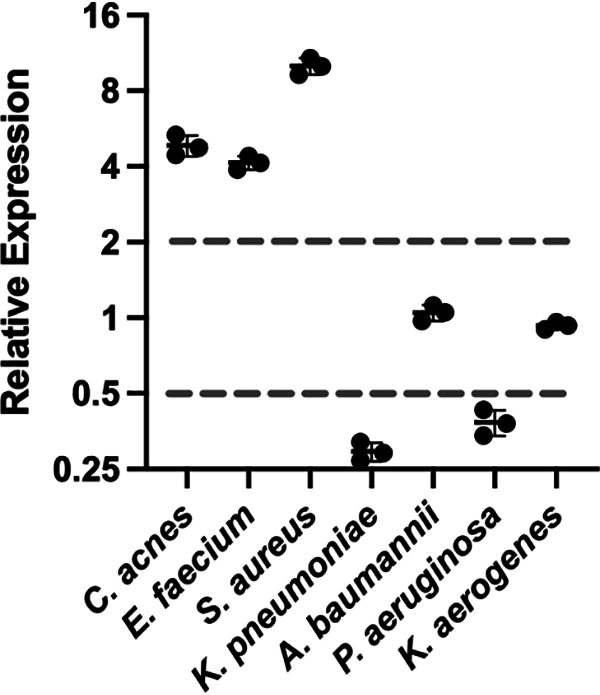
Expression profile of *clec-47* upon infection as examined by qPCR. The wild-type C. elegans N2 strain was infected with indicated bacteria for 24 h, and the total RNA was then extracted, followed by cDNA synthesis. Worms exposed to the usual laboratory food source, food E. coli strain OP50, were used as the control. Real-time PCR was performed three times to achieve 3 results of change-fold, and means ± SEM are shown.

To further investigate the role of CLEC-47 upon infection, we utilized a quantitative real-time PCR (qRT-PCR/qPCR) assay to examine the expression profile of *clec-47* in wild-type nematodes following infection with several so-called ESKAPE pathogens (Enterococcus faecium, Staphylococcus aureus, Klebsiella pneumoniae, Acinetobacter baumannii, Pseudomonas aeruginosa, and Enterobacter spp.) (28, 29). As shown in Fig. 2, compared to the usual laboratory food source (E. coli strain OP50), *clec-47* was induced more than 4-fold when nematodes were exposed to Enterococcus faecium and Staphylococcus aureus, both Gram-positive ESKAPE pathogens. Interestingly, infection with two Gram-negative ESKAPE pathogens, Klebsiella pneumoniae and Pseudomonas aeruginosa, suppressed *clec-47* expression more than 2-fold. Exposure to two other Gram-negative pathogens (the ESKAPE pathogen Acinetobacter baumannii and Klebsiella aerogenes) did not change the expression of *clec-47*. We further investigated another Gram-positive bacterial pathogen, Cutibacterium acnes. Infection with all 3 Gram-positive bacteria induced the expression of *clec-47* (Fig. 2). This expression profile indicates that CLEC-47 is regulated diversely by infection with different bacterial species, especially Gram-positive versus Gram-negative bacteria.

### CLEC-47 is a classic secretory protein with two N-glycosylation sites.

We also noticed that the segment spanning aa 7 to 29 of CLEC-47 is predicted to be a transmembrane helix by the TMHMM server (Fig. S2), which overlaps the signal peptide region (Fig. 1B). The transmembrane helix would anchor the protein in the phospholipid bilayer, which stops the process of secretion (30). To determine whether CLEC-47 is a secretory or transmembrane protein, we first expressed CLEC-47 in mammalian HEK 293 cells by transient transfection. The full-length coding sequence was inserted into plasmid pcDNA4, which is designed for eukaryotic expression. The transient expression of CLEC-47 was controlled by the human cytomegalovirus (CMV) promoter and monitored by immunoblotting against the C-terminal fusion tags (hemagglutinin [HA] tag and His tag). As shown in Fig. S4, recombinant CLEC-47–HA–His was detected in both the cell lysates and culture medium, suggesting that recombinant CLEC-47 was secreted. However, the signal for secreted CLEC-47–HA–His is weak and unstable even after we optimized the process of expression and detection. In addition, there are two bands detected in both the total cell lysate (TCL) and the supernatant (S), which might be caused by different posttranslational modifications or the nonspecific binding of anti-HA detection antibody.

10.1128/mBio.02579-21.4 FIG S4 Verification of the secretion of the CLEC-47 (CLEC-47–HA–His) in eukaryotic cells. A Western blot assay was performed to analyze the recombinant expression of CLEC-47 in HEK 293 cells. The indicated pcDNA4-HA-HIS backbone plasmids were utilized for protein expression. Six hours after transfection, the culture medium was changed to Opti-MEM I reduced-serum medium (Gibco). Immunoblotting was performed with mouse monoclonal anti-HA antibody. The data shown are representative of results from at least three independent experiments. (A) Total cell lysates; (B) supernatant (conditioned cultured medium). Download FIG S4, XLSX file, 0.1 MB. Copyright © 2021 Pan et al. 2021 Pan et al. https://creativecommons.org/licenses/by/4.0/ This content is distributed under the terms of the Creative Commons Attribution 4.0 International license.

**FIG 3:**
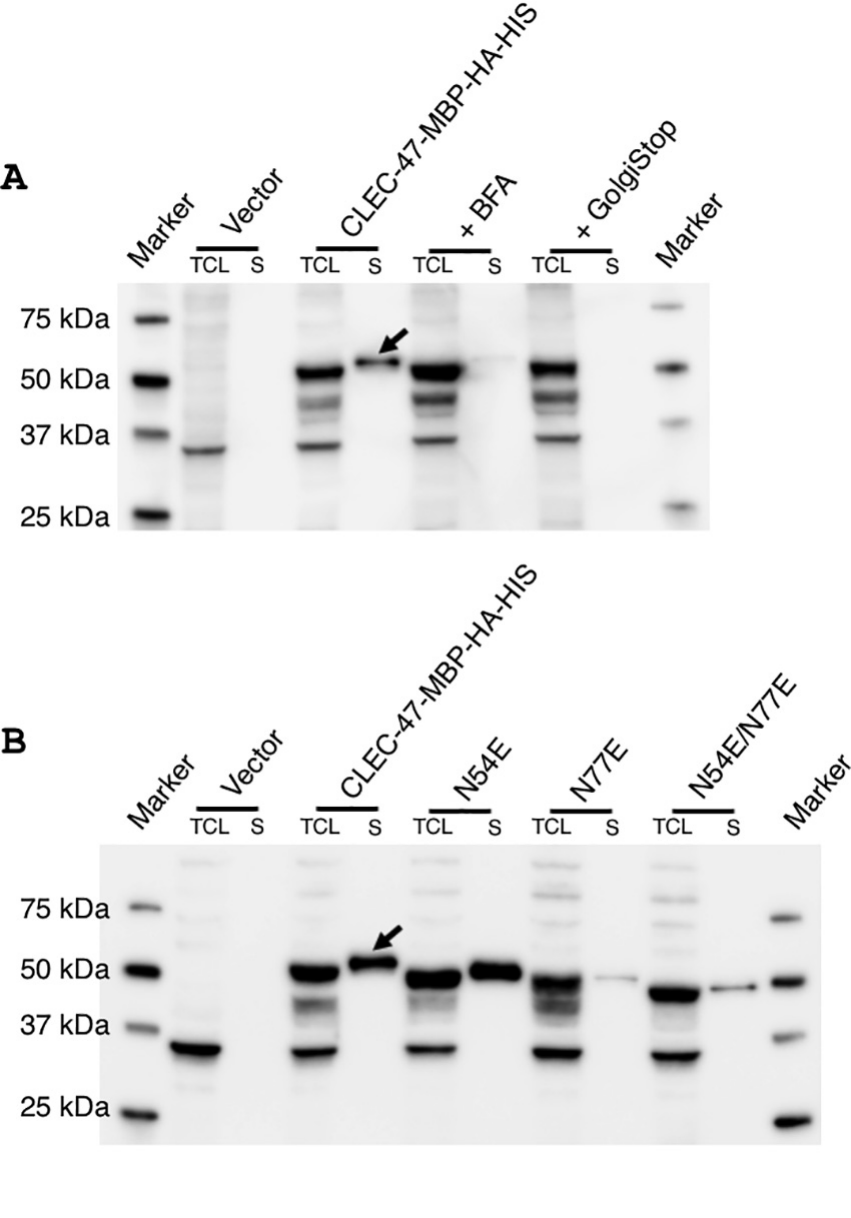
Verification of the secretion and glycosylation of CLEC-47 in eukaryotic cells. A Western blot assay was performed to analyze the recombinant expression of CLEC-47 in HEK 293 cells using a mouse anti-HA tag antibody. The indicated pcDNA4-HA-HIS backbone plasmids were utilized for protein expression. (A) CLEC-47 is secreted. Transfected cells were cultured in the absence or presence of BFA or GolgiStop for 12 h before sample collection. (B) CLEC-47 is glycosylated. Plasmids for wild-type CLEC-47 and N54E and/or N77E mutants were examined. The data shown are representative ones. Arrows indicate secretory CLEC-47.

To further explore the potential secretion of CLEC-47, we next attached a relatively large tag, maltose binding protein (MBP) (42.5 kDa), to the C terminus of CLEC-47 in addition to the HA and His tags. The theoretical molecular weight of CLEC-47–MBP–HA–His is 57.2 kDa as calculated by the ProtParam tool (https://web.expasy.org/protparam/). Using Western blotting, we found a prominent signal for CLEC-47 in the supernatant fractions (Fig. 3A and B; arrows indicate CLEC-47–MBP–HA–His). Significantly, recombinant CLEC-47–MBP–HA–His has a higher molecular weight in the supernatant fraction than in the TCL, which might be caused by posttranslational modification. Furthermore, these data indicate that the CLEC-47 signal in the supernatant is not from damaged/lysed cells since this higher-molecular-weight band is not present in the TCL. Moreover, the CLEC-47–MBP–HA–His signal in the supernatant could be blocked by the addition of brefeldin A (BFA) or GolgiStop (Fig. 3A), agents that inhibit protein transportation from the endoplasmic reticulum (ER) to the Golgi complex and within the *trans*-Golgi network. These data are consistent with the conclusion that CLEC-47 is a secreted protein.

Glycosylation is characteristic of vertebrate cytokines, and we explored the possibility that a glycosylated form of CLEC-47 is specifically secreted. As shown in Fig. S5, we found 2 N-linked glycosylation sites at N54 and N77 in CLEC-47 using the NetNGlyc server (http://www.cbs.dtu.dk/services/NetNGlyc/). We constructed two single-base-pair mutations in pcDNA4-CLEC-47, N54E and N77E, as well as the double N45E/N77E mutation and expressed the mutant forms of CLEC-47 in HEK 293 cells. When examined by Western blotting, the N54E and N77E mutants showed decreased molecular weights, and N54E/N77E demonstrated the lowest molecular weight (Fig. 3B). These results indicate that a glycosylated form of CLEC-47 is specifically secreted in eukaryotic cells.

10.1128/mBio.02579-21.5 FIG S5 Prediction of N-glycosylation sites of CLEC-47 by NetNGlyc (http://www.cbs.dtu.dk/services/NetNGlyc/). Download FIG S5, XLSX file, 0.1 MB. Copyright © 2021 Pan et al. 2021 Pan et al. https://creativecommons.org/licenses/by/4.0/ This content is distributed under the terms of the Creative Commons Attribution 4.0 International license.

### Recombinant CLEC-47 produced in a prokaryotic expression system with an MBP-His fusion tag does not inhibit bacterial growth.

**FIG 4:**
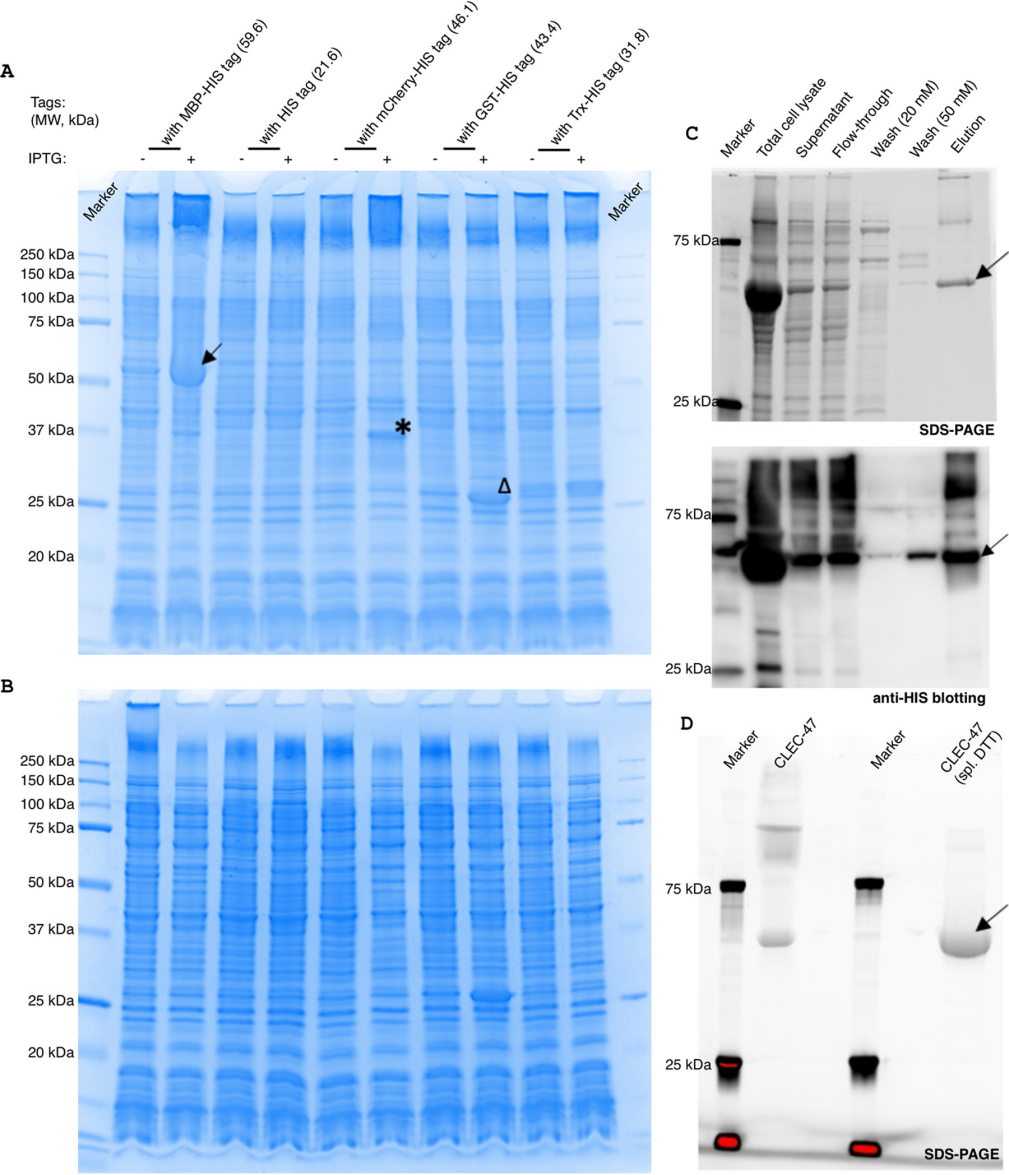
Optimization of recombinant CLEC-47 production and protein purification. (A) Total cell lysates of E. coli cells. MW, molecular weight (B) Soluble fractions of E. coli cells. CLEC-47 was fused with the indicated fusion tags. The transformed cells were cultured to an OD_600_ of 0.6 and then induced with IPTG for 3 h at room temperature. Cells were collected and lysed. The proteins were separated by SDS-PAGE. (C) Fractions from protein purification were examined by SDS-PAGE and immunoblotting with anti-His tag antibody. The arrow, star, and triangle indicate the specific bands detected. (D) Purified CLEC-47 (MBP–CLEC-47–His) (20 μg) was separated by SDS-PAGE with or without 200 mM DTT. spl., supplemental.

Because the MBP fusion tag improved the expression of recombinant CLEC-47 in eukaryotic cells, we attempted to optimize the prokaryotic expression system by testing different fusion tags (Fig. 4). Several CLEC-47 constructs were expressed in Escherichia coli cells and examined by SDS-PAGE. Fusing CLEC-47 with MBP-His resulted in readily detectable expression of CLEC-47–MBP–His in E. coli cell lysates (Fig. 4A, arrow). However, other tags (including mCherry-His tag, glutathione *S*-transferase [GST]–His tag, and thioredoxin [Trx]-His tag) did not increase the expression of CLEC-47 to levels where a clear band was visible on SDS-PAGE gels. As shown in Fig. 4A, there are some other specific bands in the samples expressing the mCherry-His and GST-His tag fusion proteins after isopropyl-β-d-thiogalactopyranoside (IPTG) induction. For example, the band marked by an asterisk displays a molecular weight of <37 kDa, which is not the recombinant CLEC-47 fusion protein (CLEC-47–mCherry–His, 46.1 kDa). Meanwhile, the triangle indicates the band with a displayed molecular weight of approximately 27 kDa, indicating that it is a recombinant GST tag only.

We further examined whether recombinant CLEC-47 (MBP–CLEC-47–His) was soluble in E. coli cells by SDS-PAGE. As shown in Fig. 4B, recombinant CLEC-47 was barely detectable in the soluble fraction. This finding indicated that the produced recombinant CLEC-47 protein has insufficient solubility. To obtain sufficient quantities of purified soluble CLEC-47 for functional studies, we scaled up the culture volume to 5 liters. MBP–CLEC-47–His was purified by immobilized-metal affinity chromatography followed by the removal of potential endotoxin by polymyxin B-agarose (Fig. 4C). As shown in Fig. 4D, purified MBP–CLEC-47–His displayed multiple bands on SDS-PAGE gels. However, after being supplied with a high concentration of dithiothreitol (DTT) (200 mM), the purified protein showed a major band with a purity of >95%. These results indicate that CLEC-47 could form polymers in solution.

**FIG 5:**
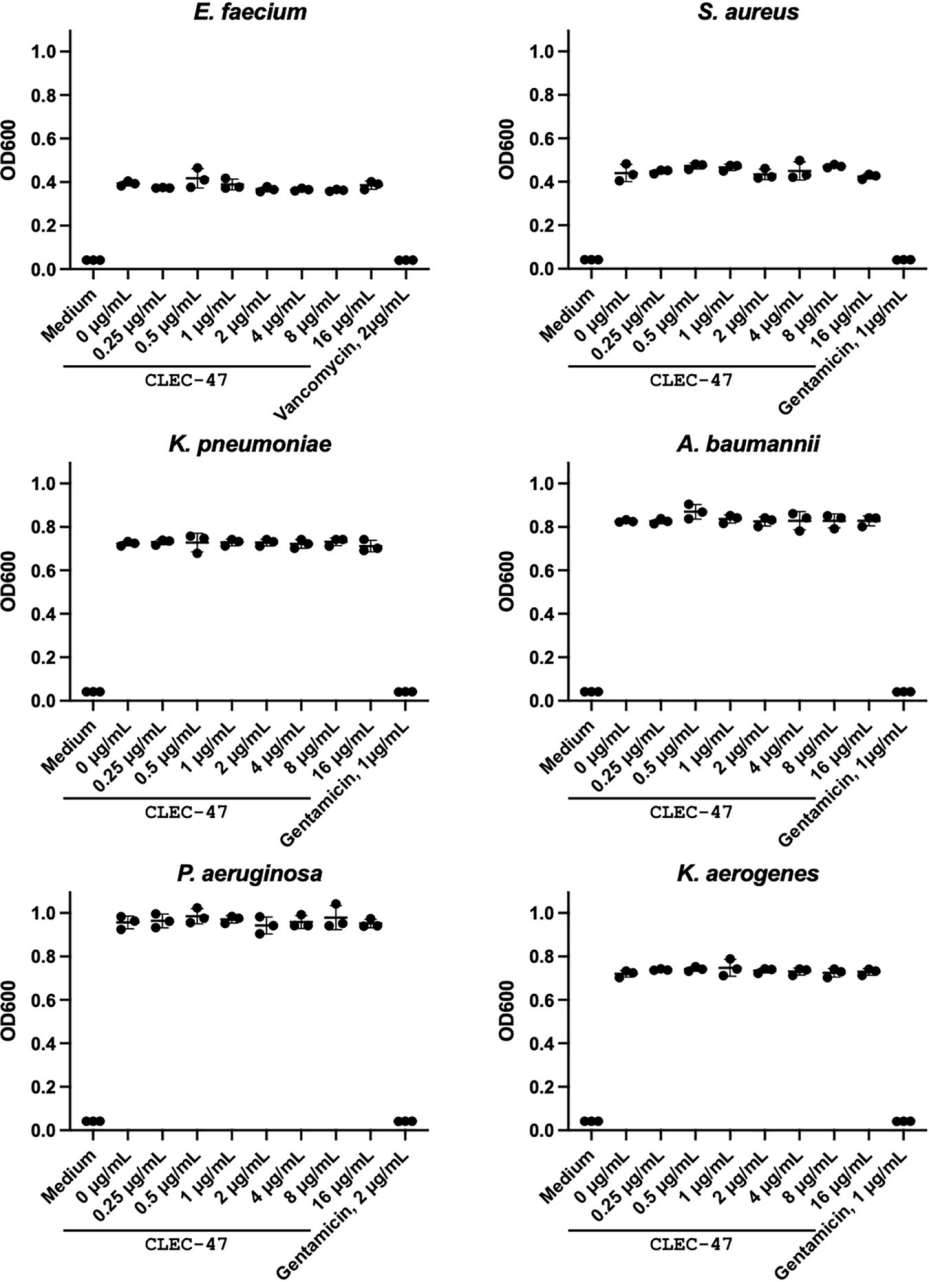
MIC assay of recombinant CLEC-47 against bacterial strains. An antimicrobial activity assay was performed in a 96-well cell culture plate in triplicate. The indicated bacteria were incubated with serial doses (micrograms per milliliter) of recombinant MBP–CLEC-47–His for 24 h. Vancomycin and gentamicin were used as the positive antimicrobial controls. The OD_600_ was measured to demonstrate cell growth.

Because *clec-47* expression in C. elegans is associated with bacterial infection, we evaluated whether the purified MBP–CLEC-47–His protein demonstrated direct antimicrobial activity. Recombinant MBP–CLEC-47–His was utilized in the MIC assay, which was performed using the broth dilution method (11, 31). However, recombinant CLEC-47 demonstrated no antimicrobial activity against E. faecium (Gram positive), S. aureus (Gram positive), K. pneumoniae (Gram negative), A. baumannii (Gram negative), P. aeruginosa (Gram negative), or K. aerogenes (Gram negative) up to the highest concentration tested (16 μg/ml) (Fig. 5).

### Recombinant CLEC-47 enhances macrophage chemotaxis with no effect on cell proliferation.

**FIG 6:**
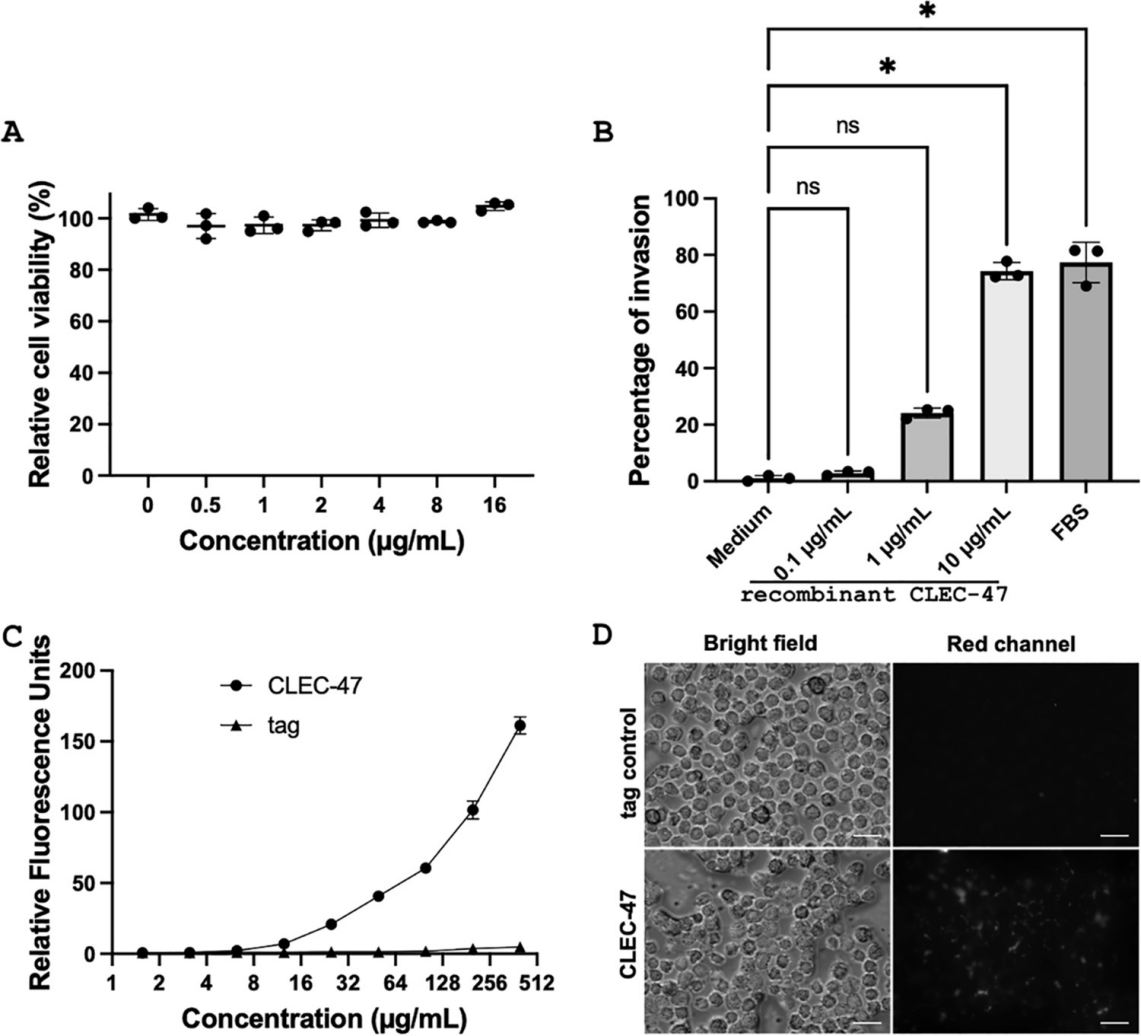
Proliferation and chemotactic effects of recombinant CLEC-47 bound to J774A.1 cells. (A) J774A.1 cells were incubated with the indicated doses of CLEC-47 for 24 h. Cell viability was monitored by adding the reagent WST-1. (B) A chemotaxis assay was performed in a set of transwell chambers at 37°C for 4 h with the indicated doses of recombinant CLEC-47. FBS (10%) was utilized as a positive control. *, *P < *0.05 by a Kruskal-Wallis multiple-comparison test; ns, not significant. (C) J774A.1 cells in a 96-well plate were washed with PBS and incubated with mCherry-tagged CLEC-47 at the indicated doses for 1 h at room temperature. The fusion tag (MBP-mCherry-His) was used as a control. (D) J774A.1 cells grown on glass coverlids were incubated with 100 μg/ml of CLEC-47 (MBP–mCherry–CLEC-47–His) or a tag control (MBP-mCherry-His) for 1 h at room temperature. Cells were washed, fixed, and loaded onto the slides for microscopy imaging. Magnification, ×400. Scale bar: 25 μm. All experiments were performed in triplicate.

Based on the evolutionary conservation of immune gene functions, we tested whether recombinant CLEC-47–MBP–His could activate immune-related responses in mammalian cells. In this series of experiments, we first investigated whether recombinant CLEC-47 was toxic to murine J774A.1 macrophage cells or could enhance cell proliferation. As shown in Fig. 6A, recombinant CLEC-47 was added at serial dosages up to 16 μg/ml. After incubation for 24 h, the proliferation of J774A.1 macrophages did not show any significant difference compared with mock-treated cells, indicating that recombinant CLEC-47 is not grossly toxic to these cells.

Next, we evaluated whether recombinant CLEC-47 was a chemoattractant for J774A.1 cells. We utilized serum as a positive control, which is a potent mixture to recruit cells (32, 33). The chemotactic ability of CLEC-47 was determined in a cell-culturing set of transwell chambers at 37°C for 4 h. CLEC-47 at 0.1 μg/ml, 1 μg/ml, and 10 μg/ml was placed in the bottom chambers, J774A.1 cells were placed in the top chambers, and the extent of migration was calculated by determining the number of J774A.1 cells that migrated from the top to the bottom chambers. As shown in Fig. 6B, 10 μg/ml of CLEC-47 induced a significant chemotactic response on J774A.1 cells, similarly to the fetal bovine serum (FBS) (10%) positive control, whereas 0.1 μg/ml of CLEC-47 did not exhibit any chemoattractant activity.

### Recombinant CLEC-47 directly binds, activates, and increases cytokine production of macrophages.

The recruitment of macrophages by a secretory protein suggests the existence of a corresponding macrophage receptor. To monitor potential CLEC-47 binding to macrophage J774A.1 cells, we inserted the coding sequence of the fluorescent mCherry protein into the E. coli plasmid expressing MBP–CLEC-47–His. Recombinant MBP–mCherry–CLEC-47–His was expressed and purified for the binding assay. As shown in Fig. 6C, after incubating J774A.1 cells with MBP–mCherry–CLEC-47–His at room temperature for 1 h, the fluorescence signal associated with the J774A.1 cells significantly increased compared to the MBP-mCherry-His tag control, suggesting that CLEC-47 directly binds to macrophage J774A.1 cells. This binding assay was confirmed by direct fluorescence microscopy imaging. As indicated in Fig. 6D, MBP–mCherry–CLEC-47–His-incubated J774A.1 cells displayed higher red fluorescence than fusion tag control (MBP-mCherry-His)-treated cells. These results shed light on the existence of a potential CLEC-47 binding partner on J774A.1 macrophages.

**FIG 7:**
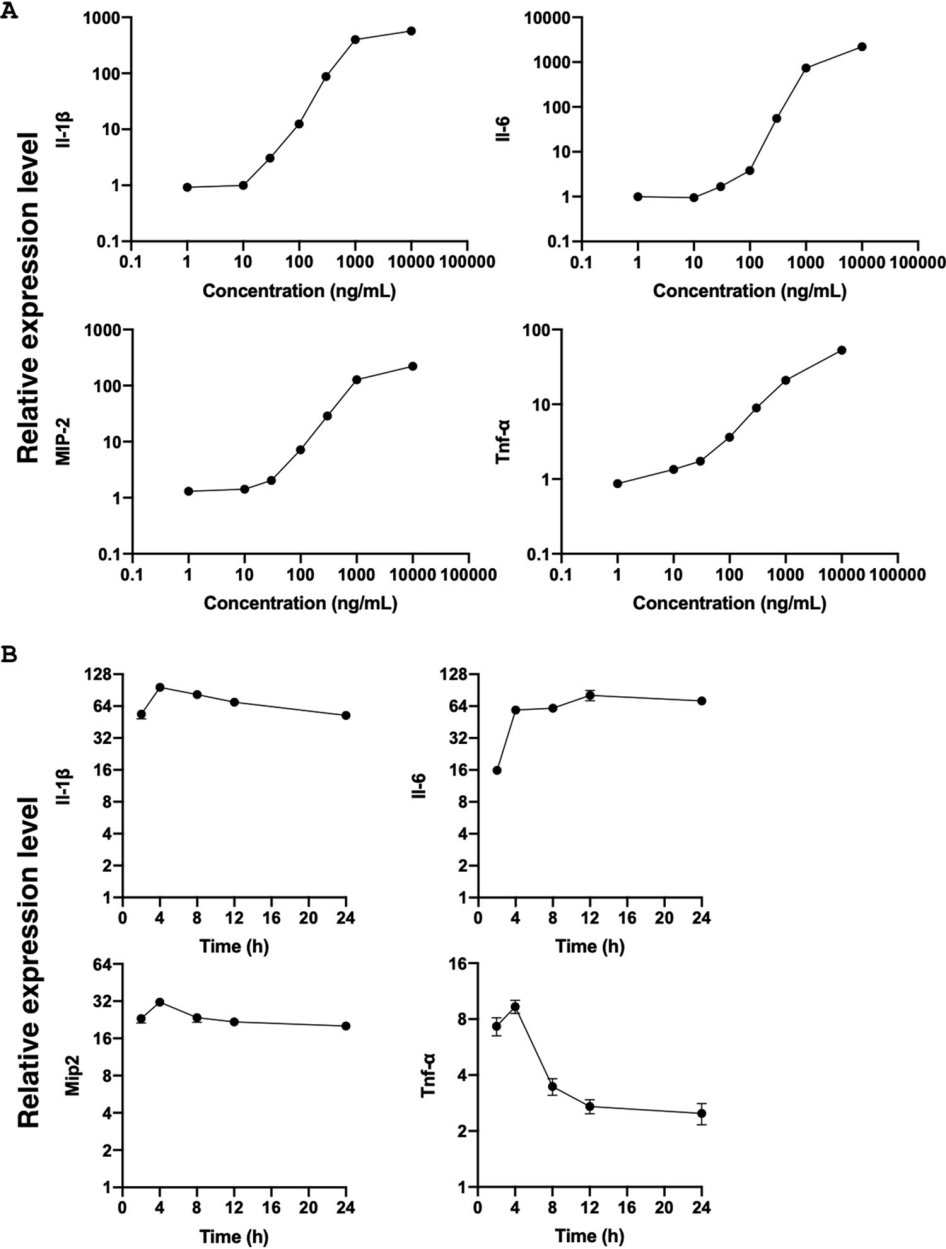
Recombinant CLEC-47 activated J774A.1 cells in dose-dependent and time-dependent manners. (A) Dose-dependent manner. J774A.1 cells were seeded into a 6-well plate and treated with the indicated concentrations of recombinant CLEC-47 protein for 6 h. The same amount of the fusion tag was utilized as an experimental control for qPCR assays. (B) Time-dependent manner. J774A.1 cells were treated with 300 ng/ml CLEC-47 or the fusion tag for the indicated times. The transcriptional levels of the indicated cytokines were normalized to that of the housekeeping gene *Hprt*. The experiments were done in triplicate, and data from representative ones are demonstrated.

Macrophages produce large amounts of cytokines after stimulation with a variety of immune-related ligands. We investigated whether incubation of macrophages with recombinant CLEC-47 resulted in the expression of genes encoding proinflammatory and/or anti-inflammatory cytokines (Fig. 7). In this series of experiments, we treated J774A.1 cells with 10 μg/ml recombinant MBP–CLEC-47–His or the same amount of the fusion tag (MBP-His) as the control (Fig. 7A) and found that the gene expression levels of TNF-α, IL-1β, IL-6, and Macrophage Inflammatory Protein 2 (MIP-2) were significantly increased. The transcriptional level of IL-10 was found to be unchanged, whereas that of IL-4 was undetectable.

In order to evaluate if the cytokine-eliciting activity of CLEC-47 is dose and time dependent, we treated J774A.1 cells with serial dilutions of recombinant MBP–CLEC-47–His (Fig. 7A). J774A.1 cells started producing detectable cytokines from a dose of recombinant CLEC-47 of 30 ng/ml, and the cytokine-stimulating effect peaked around 4 h after recombinant CLEC-47 treatment (Fig. 7B).

**FIG 8:**
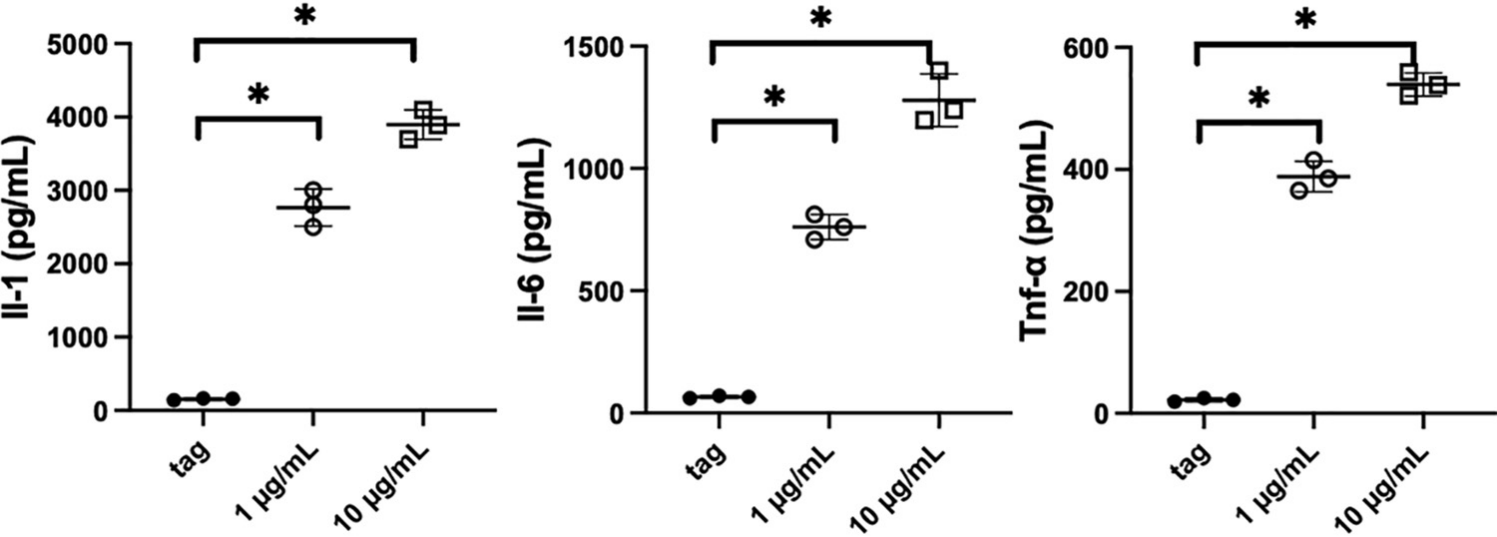
Confirmation of recombinant CLEC-47-activated J774A.1 cells by an ELISA. J774A.1 cells were treated with recombinant MBP–CLEC-47–His (1 μg/ml [circles] and 10 μg/ml [squares]) and the fusion tag (MBP-His) control (10 μg/ml [dots]) for 24 h. The supernatants were collected for analysis of cytokine levels. The experiments were performed in triplicate, and data from representative ones are demonstrated. Values are means ± standard deviations (SD). *, *P < *0.05 by ANOVA.

To confirm the results in Fig. 7B showing that CLEC-47 elicits the transcription of cytokine-encoding genes, we also measured the levels of TNF-α, IL-1β, and IL-6 using a standard enzyme-linked immunosorbent assay (ELISA). We treated J774A.1 cells with recombinant MBP–CLEC-47–His (1 μg/ml and 10 μg/ml) or with the MBP-His fusion tag (10 μg/ml) as a control for 24 h. The production of secretory cytokines in the culture media was measured, and the results, shown in Fig. 8, showed that recombinant CLEC-47 was able to activate J774A.1 macrophages to release the proinflammatory cytokines.

### Recombinant CLEC-47 directly induces the expression of antimicrobial proteins and a detoxification protein in C. elegans.

**FIG 9:**
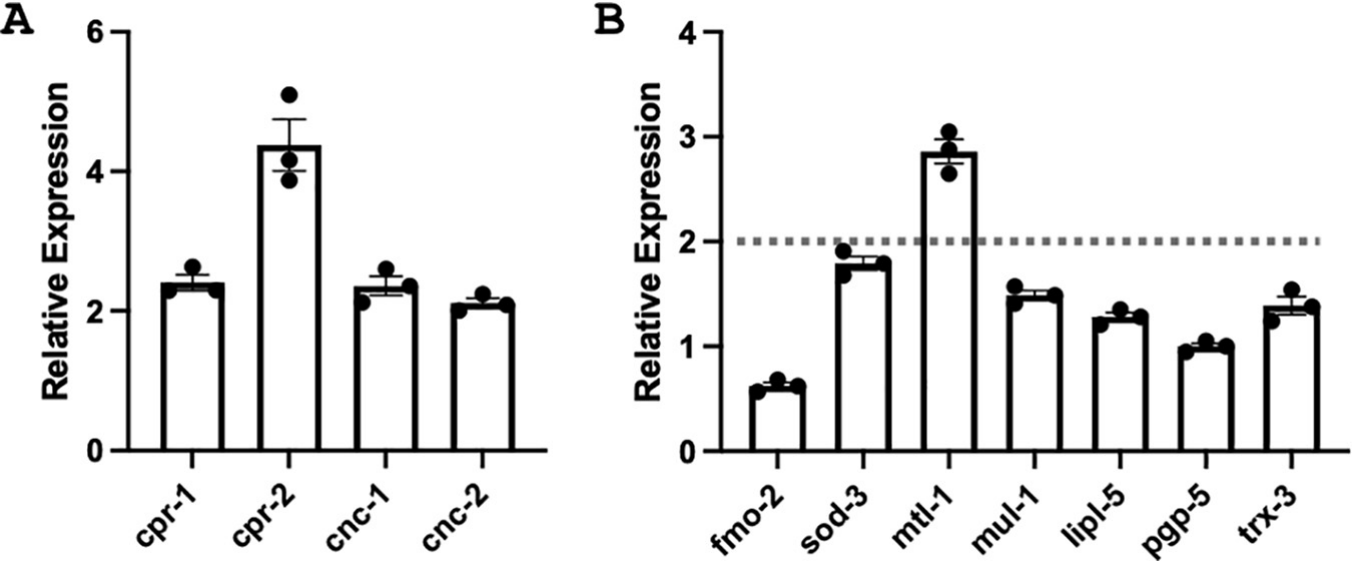
Recombinant CLEC-47 induced the transcriptional expression of antimicrobial proteins and a detoxification protein in C. elegans. (A) Induced antimicrobial proteins. (B) Detoxification proteins. Wild-type worms were fed with CLEC-47-expressing E. coli or MBP-His fusion tag-expressing cells for 24 h. Real-time PCR was performed three times to achieve 3 results of change-fold, and means ± SEM are shown.

CLEC-47 displays typical cytokine-like properties in macrophage J774A.1 cells. To further determine the *in vivo* role of CLEC-47 in C. elegans, we fed C. elegans animals with recombinant CLEC-47 (MBP–CLEC-47–His)-expressing E. coli or MBP-His fusion tag-expressing cells as a control for 24 h and monitored the expression of host immune effectors (antimicrobial peptides and proteins) and detoxification proteins by qPCR. The results for all tested effectors are listed in Table S5. As indicated in Fig. 9, among the 49 tested effectors, the expression of *cnc-1*, *cnc-2*, *cpr-1*, *cpr-2*, and *mtl-1* was increased >2-fold. *cnc-1* and *cnc-2* belong to a family of host antimicrobial effectors referred to as caenacins, whereas *cpr-1* and *cpr-2* are cysteine protease genes. *mtl-1* is a metallothionein gene that is involved in heavy metal detoxification. These observations suggest that CLEC-47 plays a direct role in the process of host defense against infection.

10.1128/mBio.02579-21.10 TABLE S5 Expression profile of antimicrobial peptides and proteins and detoxification proteins in C. elegans fed with recombinant CLEC-47-expressing E. coli cells. Download Table S5, XLSX file, 0.01 MB. Copyright © 2021 Pan et al. 2021 Pan et al. https://creativecommons.org/licenses/by/4.0/ This content is distributed under the terms of the Creative Commons Attribution 4.0 International license.

## DISCUSSION

Common features of cytokines include relatively low molecular weight, secretory expression, glycosylation, chemoattractant activity, response to an immune stimulus, and immune-modulatory activities (12). To better understand the evolutionary history of cytokines, we sought to identify cytokine-like proteins in C. elegans that function as immune mediators during bacterial infection. We found that the transcription of *clec-47* is induced by Gram-positive bacteria and suppressed by Gram-negative pathogens. Also, the CLEC-47 protein is most likely glycosylated, induces the expression of antimicrobial protein- and detoxification protein-encoding genes in C. elegans, and exhibits cytokine-like functions in mammalian cell assays. These findings suggest that nematodes express cytokine-like immune mediators, such as CLEC-47, in addition to antimicrobial peptides and proteins that have direct cytotoxic activity against pathogens.

Macrophages play a vital role in the vertebrate defense response against infection. C. elegans has specialized macrophage-like cells, referred to as coelomocytes, but each C. elegans adult animal has only 6 of them, and their role in the host defense response is unclear (34, 35). We postulated that mammalian immune cells might respond to C. elegans-encoded cytokine-like molecules such as CLEC-47. Therefore, instead of attempting to determine the role of CLEC-47 in C. elegans directly, we explored the ability of recombinant CLEC-47 to elicit immune-related responses in J774A.1 cells.

The chemotactic effect is the principal characteristic of chemokines (36). Our studies report that recombinant CLEC-47 has a chemotactic effect on mouse macrophages. This result indicates that CLEC-47 might be a chemokine-like protein in C. elegans. Chemokines are a subfamily of highly conserved cytokines in mammals, including the C, CC, CXC, and CX3C families (37). CLEC-47 contains eight cysteines (Fig. 1A) in its amino acid sequence. However, CLEC-47 does not belong to the C chemokine family, the members of which contain only two cysteines (37). Moreover, CLEC-47 does not have any CC, CXC, and CX3C motifs defined by sequentially conserved cysteine residues. Taken together, CLEC-47 lacks motifs associated with chemokine families in mammals, even though it has a chemotactic effect on mouse macrophages.

In C. elegans, genetic analysis has identified immune signaling pathways and antimicrobial peptides and proteins. For example, the C. elegans mutants *pmk-1*(*km25*), *sek-1*(*km4*), *nsy-1*(*ky400*), *tir-1*(*ok1052*), and *skn-1*(*zu67*) define components of an evolutionarily conserved p38 MAPK pathway (38). Furthermore, Zhou et al. utilized C. elegans immunity-related mutants in worm life span assays to show that the production of antimicrobial peptides and proteins (LYS-7, SPP-1, and ABF-3) defends against enterotoxigenic E. coli (39).

The posttranslational modifications are the major differences between prokaryotic and eukaryotic protein expression (40). Eukaryote-expressed proteins can undertake proteolytic cleavage (like signal peptide removal) and the addition of modifying groups such as acetyl, phosphoryl, glycosyl, and methyl (41). These modifications play important roles in protein functions. However, many commercially available recombinant cytokines were produced in the prokaryotic system (R&D Systems), and they still have relative activities. Therefore, in the current study, we chose to express recombinant CLEC-47 in a prokaryotic expression system for protein purification that has the advantage of lower cost and higher yield than HEK 293 eukaryotic cell expression systems (42, 43).

Normally, it is necessary to have a purified protein to determine the precise biological function of a novel gene that encodes a secretory protein. By utilizing a purified protein, it is possible to decipher the function of the protein directly (44). In this study, we observed that the expression of *clec-47* is highly correlated with bacterial infections, which suggests a critical role of CLEC-47 in the host immune response. We purified recombinant CLEC-47 from a prokaryotic expression system and removed endotoxin by its affinity for polymyxin B-agarose. Recombinant CLEC-47 showed significant immune-related function to activate a variety of immune-related responses in murine macrophage J744A.1 cells.

There are three forms of proteins in cells, including transmembrane proteins, secretory proteins, and cytosolic proteins (45). Transmembrane proteins are the ideal form of receptors (46), while secretory proteins usually play a role as effectors and messengers between cells (47). Signal peptides and transmembrane domains typically guide protein transport through the ER-Golgi apparatus to the cell membrane (48). CLEC-47 contains an overlapping N-terminal segment of the signal peptide and transmembrane domain, suggesting that CLEC-47 could be a secretory protein or a transmembrane protein. To determine whether CLEC-47 is secreted, we expressed full-length CLEC-47 (a CLEC-47–MBP–His fusion protein) in eukaryotic HEK 293 cells and found that CLEC-47–MBP–HA–His could be readily identified in the cell supernatants, indicating that it is a secretory protein. Moreover, we found that the ER-Golgi apparatus inhibitors BFA and GolgiStop blocked the secretion of CLEC-47, showing that secretion is Golgi apparatus dependent.

Troemel et al. found that PMK-1 regulates the expression of many infection-related effectors and, from microarray data, that *clec-47* is one of the putative immune effectors and is induced upon P. aeruginosa infection (8). We previously reported that *clec-47* played an important role in infection by C. acnes (22). In contrast to the study by Troemel et al., we found that *clec-47* was induced by 3 Gram-positive bacteria but suppressed by 2 Gram-negative pathogens, including P. aeruginosa. Our working hypothesis is that CLEC-47 is part of a specific response to Gram-positive bacteria but that some pathogens (Gram-negative bacteria in the current study) are able to suppress host immune mediators, including CLEC-47. Such phenomena have been reported in mammals. For example, Wilson et al. showed that ADP-ribosylating toxin from P. aeruginosa inhibits the synthesis of IL-1α, IL-1β, TNF-α, and IFN-γ in humans (49, 50). In addition, Bahrami et al. reported that some commensal bacteria suppress the production of IL-1α (51).

Notably, the function of a protein relies on the amino acid sequence and the three-dimensional folded structure (52). The standard technique utilized to determine the three-dimensional structure of proteins at atomic resolution is X-ray crystallography (53). To obtain protein crystals for crystallographic studies, purified recombinant CLEC-47 was premixed with different precipitants for the “hanging over vapor diffusion” method. However, no protein crystal has been observed as of the preparation of the manuscript.

In conclusion, we identified a putatively secreted C. elegans glycoprotein, CLEC-47, that can recruit, bind, and activate macrophage J774A.1 cells and induce antimicrobial-related genes (*cnc-1*, *cnc-2*, *cpr-1*, and *cpr-2*) and a detoxification gene (*mtl-1*) in C. elegans. Our data suggest that CLEC-47 may function as a novel cytokine-like signaling protein in C. elegans. Future studies will continue to resolve the protein structure of CLEC-47 and identify a putative CLEC-47 C. elegans receptor.

## MATERIALS AND METHODS

### Bioinformatic analysis.

All analyses were done according to user guides corresponding to each database, server, and local application. Gene information was obtained from the WormBase database (https://www.wormbase.org). The detailed sequences of *clec* genes were retrieved from the NCBI Nucleotide database (https://www.ncbi.nlm.nih.gov/nucleotide) by searching the gene name or reference number. The amino acid sequences were obtained from the UniProt database (https://www.uniprot.org/). Amino acid compositions and theoretical molecular weights were analyzed using the ProtParam tool (http://web.expasy.org/protparam/). The signal peptide was analyzed by the SignalP 5.0 server (http://www.cbs.dtu.dk/services/SignalP/). The transmembrane domain was predicted by the TMHMM server (http://www.cbs.dtu.dk/services/TMHMM/). The evolutionarily conserved domain was examined with a Web-based tool, SMART (http://smart.embl-heidelberg.de). To clone the genes of interest, the restriction enzyme digestion sites were analyzed with a local application named EnzymeX from Nucleobytes. PCR primers were designed in EnzymeX and verified with the NCBI Primer-BLAST server (https://www.ncbi.nlm.nih.gov/tools/primer-blast/).

### Bacterial strains and culture method.

Enterococcus faecium isolate E007 and Cutibacterium acnes strain ATCC 6919 were maintained in brain heart infusion (BHI) broth (BD, Franklin Lakes, NJ, USA). Staphylococcus aureus strain MW2 was grown in tryptic soy broth (TSB) (BD, Franklin Lakes, NJ, USA). Klebsiella pneumoniae strain WGLW2, Acinetobacter baumannii strain ATCC 17978, Pseudomonas aeruginosa strain PA14, Klebsiella aerogenes strain ATCC 13048, and Escherichia coli were cultured in Luria broth (LB) (BD, Franklin Lakes, NJ, USA). All bacteria were grown at 37°C.

### Eukaryotic cell culture.

Cells were purchased from the American Type Culture Collection and maintained in base medium supplemented with 10% fetal bovine serum (FBS) at 37°C with 5% CO_2_ in the atmosphere. Murine macrophages (J774A.1 cell line) were cultured in Dulbecco’s modified Eagle’s medium (DMEM). Human embryonic kidney epithelial HEK 293 cells were cultured in ATCC-formulated Eagle’s minimum essential medium.

### Cytokine production.

Conditioned culture media were collected at the indicated time points and centrifuged to remove cell debris. All samples were aliquoted and stored at −80°C before being tested. The protein levels of murine cytokines (TNF-α, IL-1β, and IL-6) were determined by utilizing commercial sandwich enzyme-linked immunosorbent assay (ELISA) kits (BioLegend, San Diego, CA, USA). All procedures were performed according to the manufacturer’s recommendations. The experiments were performed in triplicate wells and repeated three times.

### Nematode culture.

The wild-type C. elegans N2 strain used in the current study was acquired from the Caenorhabditis Genetics Center (CGC) (University of Minnesota, MN, USA). Worms were reared and maintained at 16°C on nematode growth medium (NGM) agar plates with heat-killed E. coli strain OP50 as the food source unless otherwise specified. Age-synchronized L4 larval or young adult worms were used for all experiments.

### RNA extraction and cDNA synthesis.

After washing with phosphate-buffered saline (PBS), nematodes were harvested and homogenized using cycles of freezing and thawing in liquid nitrogen. Mammalian cells were washed with cold PBS and collected. The worms and cells were lysed in TRIzol reagent (Invitrogen) according to the manufacturer’s instructions. Chloroform and isopropanol were applied to separate total RNA. Next, RNA was washed in 70% ethanol and dissolved in RNase-free water. The quality and quantity of nucleic acids were determined by measuring the absorbance at 260 nm with a NanoVue UV-visible spectrophotometer (GE, Boston, MA). Potential contaminating genomic DNA was removed by treatment with the Turbo DNA-free kit (Invitrogen). Single-stranded cDNA was synthesized using a Verso cDNA synthesis kit (Thermo Fisher Scientific, Waltham, MA) according to the manufacturer’s instructions.

### Analysis of gene expression by real-time PCR.

iTaq universal SYBR green supermix was utilized for qPCR in a CFX96 Touch real-time PCR detection system (Bio-Rad) with the following protocol: an initial denaturation step at 95°C for 30 s, followed by 40 cycles of 95°C for 5 s and 60°C for 30 s and then ending by a melt-curve analysis from 65°C to 95°C with the instrument default setting. The primers are listed in Table S4 in the supplemental material. Gene expression was normalized using the housekeeping genes *snb-1*, *actin*, and *ama-1* for C. elegans and *Hprt* for mice. Fold changes were calculated using the 2^−ΔΔ^*^Cq^* method and compared to the expression levels from the control group. Assays were performed with three independent experimental samples in triplicate. Differences in gene expression were assessed using one-way analysis of variance (ANOVA) followed by Dunnett’s multiple-comparison test. A *P* value of <0.05 was considered statistically significant.

10.1128/mBio.02579-21.9 TABLE S4 qPCR primers used in the expression profile assay. Download Table S4, XLSX file, 0.01 MB. Copyright © 2021 Pan et al. 2021 Pan et al. https://creativecommons.org/licenses/by/4.0/ This content is distributed under the terms of the Creative Commons Attribution 4.0 International license.

### Plasmid construction.

Protein-coding segments were cloned from the cDNA library by conventional PCR. At the 5′ and 3′ termini of each gene, 20 nucleotides were synthesized and used as primers. The second set of primers with sequences of the indicated restriction enzymes was utilized to insert gene segments into plasmid pET-51b (Table 2). Mutagenesis PCR was used for inserting coding segments into plasmid pcDNA4 (a gift from Elena Oancea, Brown University). Several fusion tags (HA, His_6_, MBP, GST, Trx, and mCherry) were also inserted into the plasmids at the indicated positions by mutagenesis PCR. Moreover, single-site mutations were constructed by mutagenesis PCR. Q5 high-fidelity 2× master mix (New England BioLabs [NEB], Ipswich, MA, USA) was used for PCR. A PureLink quick gel extraction and PCR purification combo kit and a PureLink HiPure plasmid miniprep kit (Invitrogen) were used to recover DNA and extract plasmids from E. coli. One Shot TOP10 chemically competent E. coli cells from Invitrogen were utilized for the transformation procedure. The sequences of DNA segments and constructed plasmids were confirmed by sequencing, done by Genewiz (Cambridge, MA, USA).

### Secretion confirmation.

The heterologous HA-His- or MBP-HA-His-tagged protein CLEC-47 from C. elegans was expressed in HEK 293 cells, and secretion was confirmed by immunoblotting. Cells were seeded into a 6-well plate 1 day before transfection. pcDNA4-based plasmids were transfected with Lipofectamine 3000 reagent (Invitrogen, Carlsbad, CA, USA). The medium was replaced with Opti-MEM I reduced-serum medium (Gibco, Thermo Fisher Scientific, Inc., Waltham, MA, USA) at 6 h posttransfection. Cells were collected and lysed with radioimmunoprecipitation assay (RIPA) lysis and extraction buffer (Thermo Fisher Scientific).

Recombinant proteins in total cell lysates and conditioned culture medium were separated on an 8% to 16% Mini-Protean TGX stain-free protein gel (Bio-Rad, Hercules, CA, USA) and transferred to a polyvinylidene difluoride (PVDF) membrane (Bio-Rad), which was blocked with a membrane-blocking solution (Invitrogen). HA tag monoclonal antibody (12CA5) (Invitrogen) was utilized for detection. The membrane was developed with an enhanced chemiluminescence (ECL) system, and images were acquired with ChemiDoc imaging systems (Bio-Rad).

The inhibition of secretion was performed by adding manufacturer-recommended amounts of brefeldin A (BFA) or GolgiStop (containing monensin) (BD Biosciences, San Jose, CA, USA) to the changed Opti-MEM I reduced-serum medium.

### Expression and purification of prokaryotic protein in E. coli cells.

The recombinant plasmid was transformed into E. coli BL21-CodonPlus(DE3)-RIPL cells for protein expression. The cells were cultured in LB medium with antibiotics (34 μg/ml chloramphenicol and 100 μg/ml ampicillin) at 37°C overnight. The next day, the culture was diluted 100-fold in 625 ml of fresh medium in a 2-liter flask and shaken at 37°C until the optical density at 600 nm (OD_600_) of the culture reached 0.6. The culture temperature was then decreased to room temperature (∼20°C). Next, the cells were incubated in the presence of 0.1 mM isopropyl-β-d-thiogalactopyranoside (IPTG), and induction was completed at room temperature overnight (54, 55). On the third day, the cells were harvested by centrifugation at 4,000 × *g* for 10 min at 4°C and washed with cold PBS once.

For protein purification, cells were resuspended in buffer A (20 mM Tris-HCl, 100 mM NaCl [pH 8.0]) in the formation of 10 ml buffer per g of cells and lysed by sonication using a Branson 450 sonifier (output control, 2; duty cycle, 30%; time period, 15 min) in the presence of 5 μg/ml lysozyme. After sonication, the homogenized E. coli cells were separated by centrifugation at 10,000 × *g* for 60 min at 4°C to remove the debris. Next, the cleared soluble partition was filtered with a 0.45-μm filter and loaded onto an equilibrated Ni-nitrilotriacetic acid (NTA) resin column. After washing with buffer containing 20 mM or 50 mM imidazole, the recombinant protein was eluted with buffer containing a higher concentration of imidazole (300 mM). The potential endotoxin in the elution fraction was removed by flowing through polymyxin B-agarose. Finally, the purified protein was buffer changed with sterile PBS and concentrated with Amicon Ultra centrifugal filters. The final concentration of purified protein was determined with a Pierce bicinchoninic acid (BCA) protein assay kit and confirmed with a NanoVue UV-visible spectrophotometer. Recombinant protein was aliquoted and stored at −80°C.

### MIC assay.

The antimicrobial activity of recombinant protein *in vitro* was determined using the broth dilution method (11, 31). Bacteria were cultured overnight and diluted to 5 × 10^5^ CFU/ml. The recombinant protein was 2-fold diluted, and 50 μl of bacteria was transferred to a 96-well tissue culture plate. Another 50 μl of bacteria was added. The plate was incubated at 37°C for 24 h. The growth of bacteria was measured as the OD_600_ at room temperature using a spectrophotometer (SpectraMax M2; Molecular Devices, Sunnyvale, CA, USA). The assay was conducted in triplicate.

### Chemotaxis of J774A.1 cells.

The chemotactic ability of recombinant CLEC-47 protein was assayed in a 24-well plate with Boyden chamber inserts with 5.0-μm pores (catalog no. ab235696; Abcam, Cambridge, MA, USA). Cell migration was analyzed directly by reading the fluorescence (excitation [Ex]/emission [Em] wavelength of 530/590 nm). Briefly, J774A.1 cells were prewashed and resuspended in DMEM without supplementation with FBS. The cell density was set to 1 × 10^6^ cells/ml. The bottom chambers were filled with 600 μl of DMEM with different concentrations of recombinant protein. The top chambers were filled by adding 200 μl of the cell suspension. The assay plate was incubated at 37°C in a CO_2_ incubator for the indicated time (4 h). The invasive cells were separated, stained, and quantified according to the manufacturer’s instructions. Meanwhile, serially diluted J774A.1 cells were applied to determine the standard curve of the cell number by quantifying the fluorescence signal. The assay was conducted in triplicate. The percentage of invasion was calculated as (cell number in the bottom chamber)/(total cell number added to the top chamber) × 100.

### Cell viability assay.

The cells were plated at a density of 1 × 10^6^ cells/ml with 50 μl per well into a 96-well tissue culture plate. The recombinant protein was 2-fold diluted with the cell culture medium. Serial dilutions of recombinant protein were added to each well of the 96-well plate. Next, the plate was incubated at 37°C for 20 h. After adding 10 μl of the cell proliferation reagent WST-1 (Roche, Mannheim, Germany) to each well, the plate was incubated for another 4 h. The absorbance at 450 nm was monitored with a reference wavelength of 630 nm. The percent relative cell viability in each group was calculated by comparison to that of the control group.

### Examination of protein expression in E. coli.

Transformed BL21(DE3) or BL21-CodonPlus(DE3)-RIPL cells were cultured in LB medium with antibiotics overnight. The next day, the culture was diluted 100-fold in 4 ml of fresh medium. IPTG was added when the OD_600_ of the culture reached 0.6. The induction of recombinant protein was performed under the indicated conditions of time and temperature. One milliliter of the culture at an OD of 1 was pelleted, followed by lysis in 50 μl B-Per bacterial protein extraction reagent (Thermo Scientific). The total cell lysate and soluble partition were separated on an 8% to 16% Mini-Protean TGX stain-free protein gel. Next, the gel was stained with SimplyBlue SafeStain (Invitrogen).

### Recombinant protein binding assay.

The monomer tag mCherry was inserted into MBP–CLEC-47–His_10_ by mutagenesis PCR, which encoded the MBP–mCherry–CLEC-47–His_10_ protein. The recombinant protein was expressed in BL21-CodonPlus(DE3)-RIPL cells and purified by immobilized-metal affinity chromatography with Ni-NTA resin followed by polymyxin B-agarose for endotoxin removal. Next, J774A.1 cells were cultured in a 96-well plate for 24 h. The culture medium was removed, followed by 3 washes of cells. The recombinant protein or fusion tag control was added to cells for incubation for 1 h at room temperature. The plate was washed, and the fluorescence was monitored using a SpectraMax M2 spectrophotometer.

### Fluorescence microscopy imaging.

J774A.1 cells were seeded into a 24-well cell culture plate with glass coverlids inside. Three days later, the coverlids were transferred to new wells and washed with PBS. Next, 200 μl of the MBP–mCherry–CLEC-47–His fusion protein was added, and the mixture was incubated for 1 h at room temperature. After three washes, the coverlids were mounted onto glass slides for imaging. A Nikon Eclipse 80i microscope was utilized at a magnification of ×400. Images were taken with a Q-Imaging Retiga EXi FAST1394 camera.

### Statistical analysis.

All the statistical analyses were performed using GraphPad Prism 9 (GraphPad Software, San Diego, CA, USA). Bars in each figure represent the means ± standard errors of the means (SEM). One-way analysis of variance (ANOVA) followed by Dunnett’s test was used to determine the differences among three or more groups, whereas an unpaired *t* test was used if the comparison was performed between two groups. The level of statistical significance was set at a *P* value of <0.05.
